# A case report of J wave syndrome with abnormal potentials in both right and left ventricles and reversed J wave in lead V1

**DOI:** 10.1093/ehjcr/ytag078

**Published:** 2026-02-03

**Authors:** Yasuyuki Takada, Junichi Kamoshida, Muryo Terasawa, Kazuhiro Satomi, Yoshinao Yazaki

**Affiliations:** Department of Cardiology, Tokyo Medical University, 6-7-1 Nishi-shinjuku, Shinjuku-ku, Tokyo 160-0023, Japan; Department of Cardiology, Tokyo Medical University, 6-7-1 Nishi-shinjuku, Shinjuku-ku, Tokyo 160-0023, Japan; Department of Cardiology, Tokyo Medical University, 6-7-1 Nishi-shinjuku, Shinjuku-ku, Tokyo 160-0023, Japan; Department of Cardiology, Tokyo Medical University, 6-7-1 Nishi-shinjuku, Shinjuku-ku, Tokyo 160-0023, Japan; Department of Cardiology, Tokyo Medical University, 6-7-1 Nishi-shinjuku, Shinjuku-ku, Tokyo 160-0023, Japan

**Keywords:** Case report, Ventricular fibrillation, J wave syndrome, Brugada syndrome, Early repolarization syndrome, Epicardial mapping

## Abstract

**Background:**

Abnormal epicardial potentials in J wave syndrome predominantly involve the right ventricular outflow tract (RVOT), while left ventricular (LV) involvement remains less characterized and associates with increased ventricular fibrillation (VF) risk. We report a case demonstrating an inverted J wave in lead V1, suggesting LV posterior wall substrate rather than typical RVOT involvement.

**Case summary:**

A 19-year-old man with resuscitated VF received a subcutaneous implantable cardioverter-defibrillator (S-ICD). Despite cilostazol and quinidine therapy, he experienced five appropriate shocks within 6 months. Twelve-lead electrocardiography showed inferior J waves and a negative deflection in V1, suggesting an inverted J wave. Epicardial mapping showed fractionated potentials in both RVOT and LV posterior wall. Pilsicainide administration augmented RVOT potentials while attenuating those in the LV posterior wall. Spontaneous VF was triggered by premature ventricular contractions (PVCs) originating from the LV posterior wall, where prepotentials preceded QRS onset by 50 ms. Radiofrequency applications eliminated the PVCs, followed by anatomical ablation of RVOT fractionated regions. Subsequently, VF was never induced by program stimulation of up to triple extra stimuli. In the post-operative electrocardiogram, inverted J waves in the V1 lead *d*isappeared. At 8 months post-ablation, the patient remained free from VF recurrence without antiarrhythmic medications, and no S-ICD therapies occurred.

**Discussion:**

This case demonstrates complex electrophysiological manifestations of J wave syndrome, with the inverted J wave in V1 potentially reflecting LV posterior wall substrate. Although the overlapping ablation procedure limited definitive attribution, these findings contribute to understanding the heterogeneous substrates in J wave syndrome.

Learning points

- Reversed J-waves in lead V1 may reflect arrhythmogenic substrate of the left ventricular posterior wall among patients with J-wave syndrome.
- Comprehensive epicardial mapping can reveal multiple arrhythmogenic substrates across right and left ventricles that respond differently to sodium channel blockers.

## Introduction

J wave syndrome is an inherited ion channelopathy encompassing Brugada syndrome and early repolarization syndrome (ERS), causing sudden cardiac death from ventricular fibrillation (VF).^1^ Catheter ablation targeting abnormal epicardial potentials in the right ventricular outflow tract (RVOT) effectively suppresses arrhythmic events.^2^ Abnormal epicardial potentials corresponding to arrhythmogenic substrates have recently been identified on the left ventricular (LV) side, although LV involvement is less frequently reported and is associated with increased VF risk.^3^

We describe a rare case of recurrent VF in a young patient where an inverted J wave in V1 corresponded to a posterior LV wall substrate rather than typical RVOT involvement. The contrasting effects of pilsicainide on RVOT and LV electrograms, combined with successful ablation outcomes, provide new mechanistic and therapeutic insights into J wave syndrome.

## Summary figure

Clinical course and electrophysiological findings. Timeline showing clinical events from initial sudden cardiac arrest with VF to recurrent appropriate S-ICD shocks despite medical therapy, leading to catheter ablation. Pre- and post-ablation 12-lead electrocardiogram demonstrating persistent inferior J waves but resolution of the inverted J wave in lead V1 (arrow in inverted V1 lead). Epicardial maps showing abnormal potentials (dots) and ablation sites.

**Figure:**
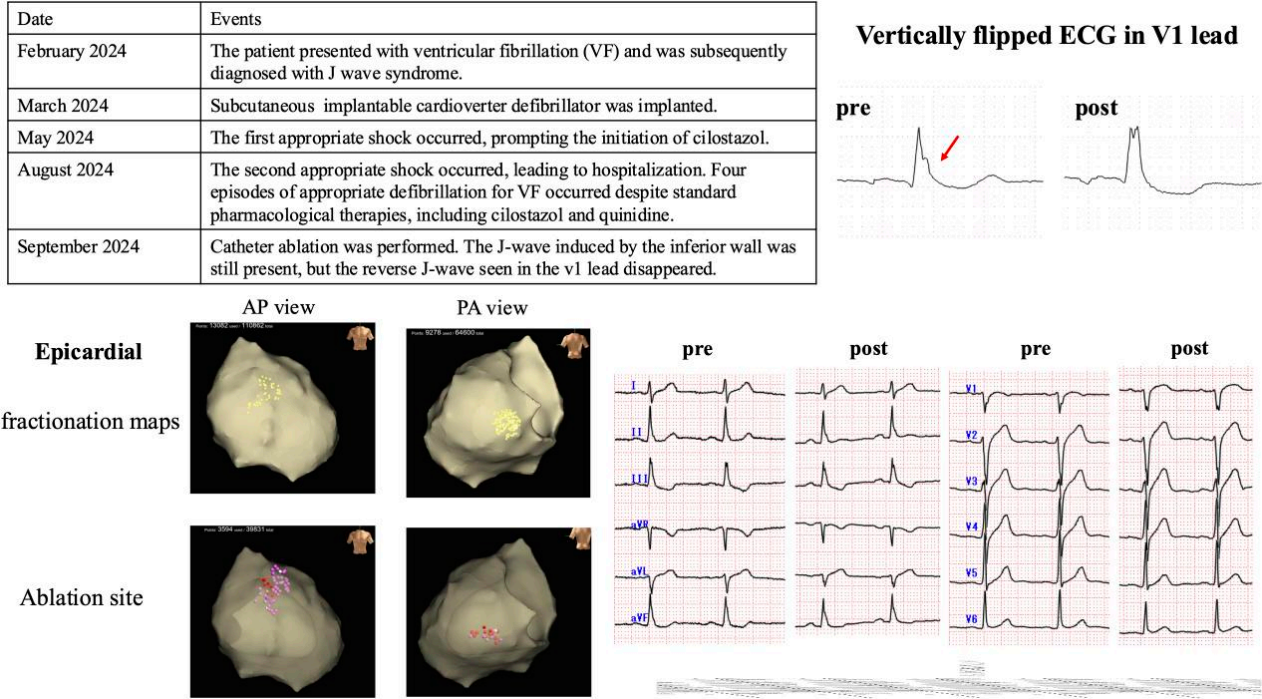


## Case presentation

A 19-year-old man without a significant past history was found unresponsive at home. Bystander CPR was initiated by his parents. Upon the arrival of emergency medical services, the initial rhythm was recorded as VF. After one defibrillation attempt, the rhythm converted to asystole. Administering 1 mg epinephrine and cardiopulmonary resuscitation restored spontaneous circulation and breathing.

**Figure 1:**
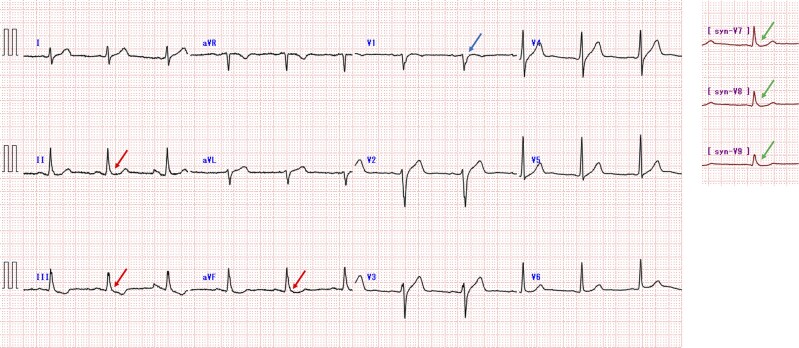
12-Lead electrocardiogram including synthesized posterior leads (syn-V7, syn-V8, syn-V9) was conducted when the patient was admitted to the hospital. The arrows indicate the J wave in the inferior wall lead and synthesized posterior leads, and inverted J wave in the V1 lead.

The patient was intubated and underwent targeted temperature management for 2 days. He was successfully extubated without any major neurological sequelae. Blood tests showed no significant abnormalities, including normal cardiac biomarkers and troponin levels. Chest radiography, echocardiography, and whole-body computed tomography, including coronary artery, were unremarkable. A 12-lead electrocardiogram (ECG) showed sinus rhythm with prominent J waves in the inferior leads, and a negative component following the QRS complex was observed in the V1 lead. Mathematically derived posterior leads (V7–V9) demonstrated J wave elevation (*Figure 1*).

Pilsicainide provocation tests revealed no prominent Brugada or ERS patterns. Signal-averaged electrocardiography showed no late potentials. Subcutaneous implantable cardioverter-defibrillator (S-ICD) implantation was performed for secondary prevention. Cardiac magnetic resonance imaging was not performed due to expected S-ICD-related artefacts compromising myocardial tissue assessment. Genetic testing for inherited arrhythmia syndromes was negative. Over 6 months, the patient experienced five appropriate S-ICD discharges for VF triggered by monomorphic premature ventricular contractions (PVCs) despite cilostazol (100 mg/day) and quinidine (200 mg/day). Holter monitoring showed only rare PVCs (4 beats/day). Due to recurrent VF on medical therapy, catheter ablation was therefore undertaken.

**Figure 2:**
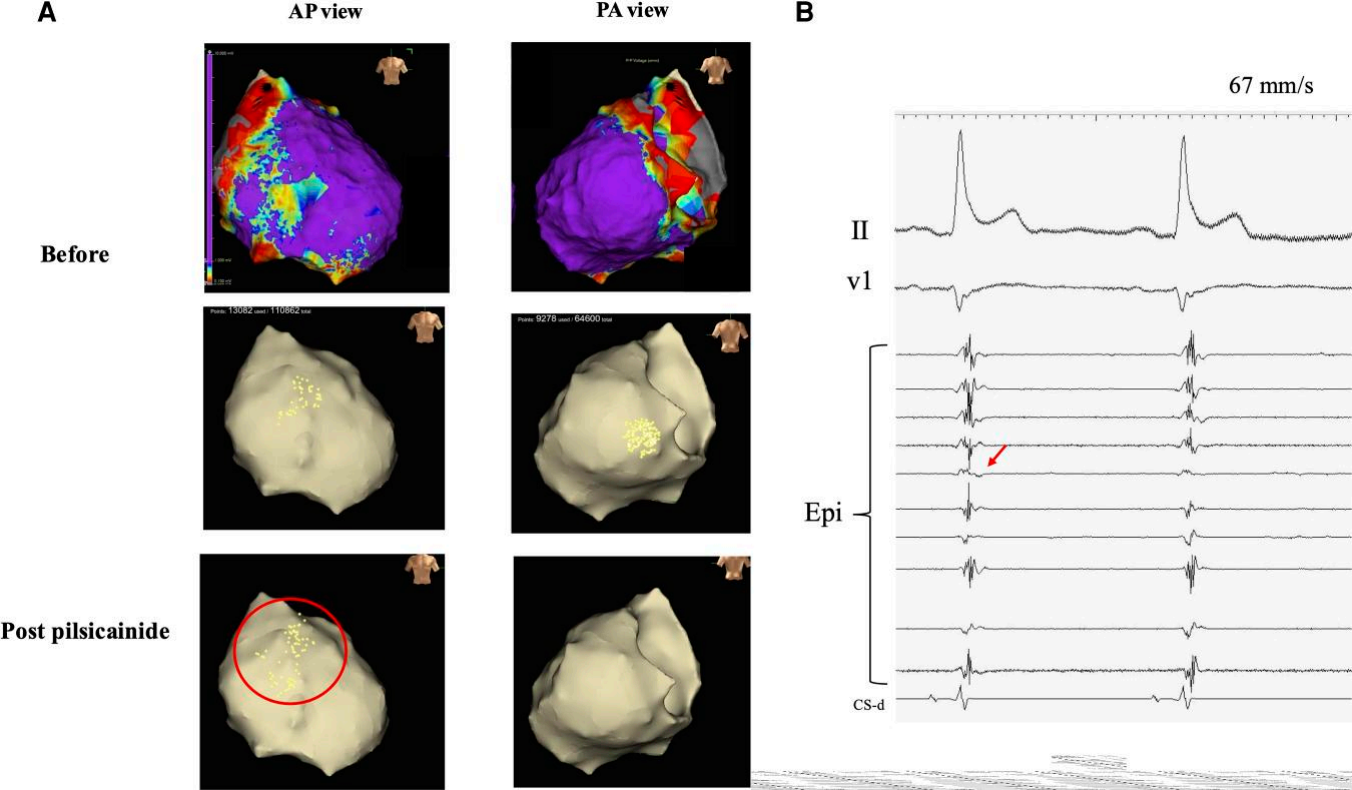
(*A*) Epicardial voltage map and fractionation maps before and after pilsicainide administration. Low voltage areas (<1.0 mV) were observed adjacent to the right ventricular free wall. Abnormal potentials are indicated by dots. After drug administration, the abnormal potentials in RVOT expanded, as highlighted by the circles. (*B*) Fragmented potentials coinciding with J wave within RVOT substrate (indicated by arrows). RVOT, right ventricular outflow tract.

The ablation was performed under deep sedation with propofol and fentanyl. Vascular access included right jugular and bilateral femoral approaches. Following coronary angiography, epicardial access was achieved via a subxiphoid approach under echocardiographic guidance. Mapping was performed using an HD-Grid catheter (Abbott) and a three-dimensional mapping system (Ensite™ NavX™, Abbott). Endocardial mapping revealed no low-voltage areas (<1.5 mV) or abnormal potentials. Epicardial examination showed low-voltage areas (<1.0 mV) adjacent to the right ventricular free wall. Abnormal potentials defined as low-amplitude (<1.0 mV) fractionated electrograms with prolonged duration (>80 ms) were identified in RVOT and the LV posterior wall. Decrement evoked potential mapping with right ventricular pacing showed no worsening of abnormal potentials. After pilsicainide (1 mg/kg) administration, RVOT abnormal potentials became apparent, whereas those in the LV posterior wall decreased (*Figure 2*).

**Figure 3:**
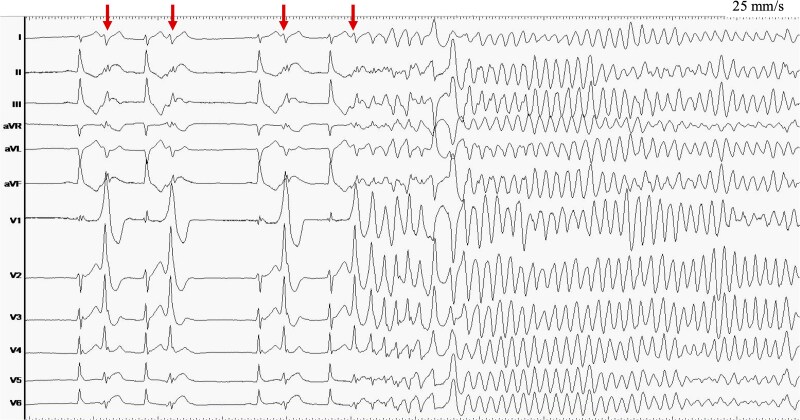
Ventricular fibrillation was spontaneously induced from monomorphic premature ventricular contraction during mapping (arrows).

During mapping, monomorphic PVCs triggered VF, requiring external defibrillation (*Figure 3*).

**Figure 4:**
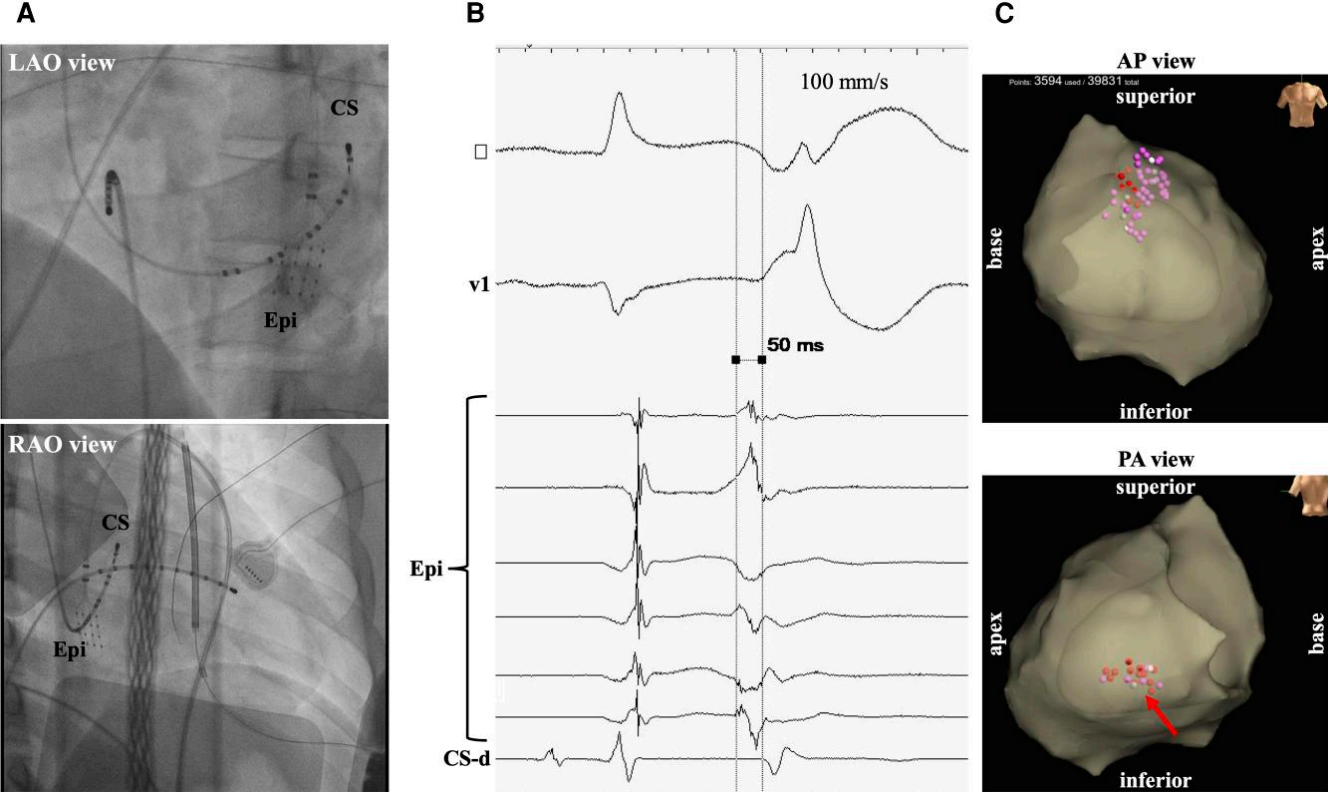
(*A*) X-ray imaging during premature ventricular contraction cauterization. (*B*) The local prepotential at the ablation point was 50 ms ahead of the premature ventricular contraction. (*C*) Ablation site indicated by three-dimensional mapping. The arrow shown on the left ventricle posterior wall corresponds to the earliest site of premature ventricular contraction.

Given frequent monomorphic PVCs, we initially targeted this arrhythmic focus. Detailed activation mapping using the HD-Grid catheter localized the earliest activation site to the LV posterior wall, where prepotentials preceded QRS onset by 50 ms. Pace mapping yielded excellent morphological concordance (12/12 leads) with the clinical PVC, confirming the origin. Prepotential characteristics suggested local tissue abnormalities consistent with fractionated electrograms in this region. Radiofrequency ablation successfully eliminated the PVCs. We subsequently performed additional ablation in RVOT regions exhibiting abnormal potentials, considering these as a potential arrhythmogenic substrate (*Figure 4*).

**Figure 5:**
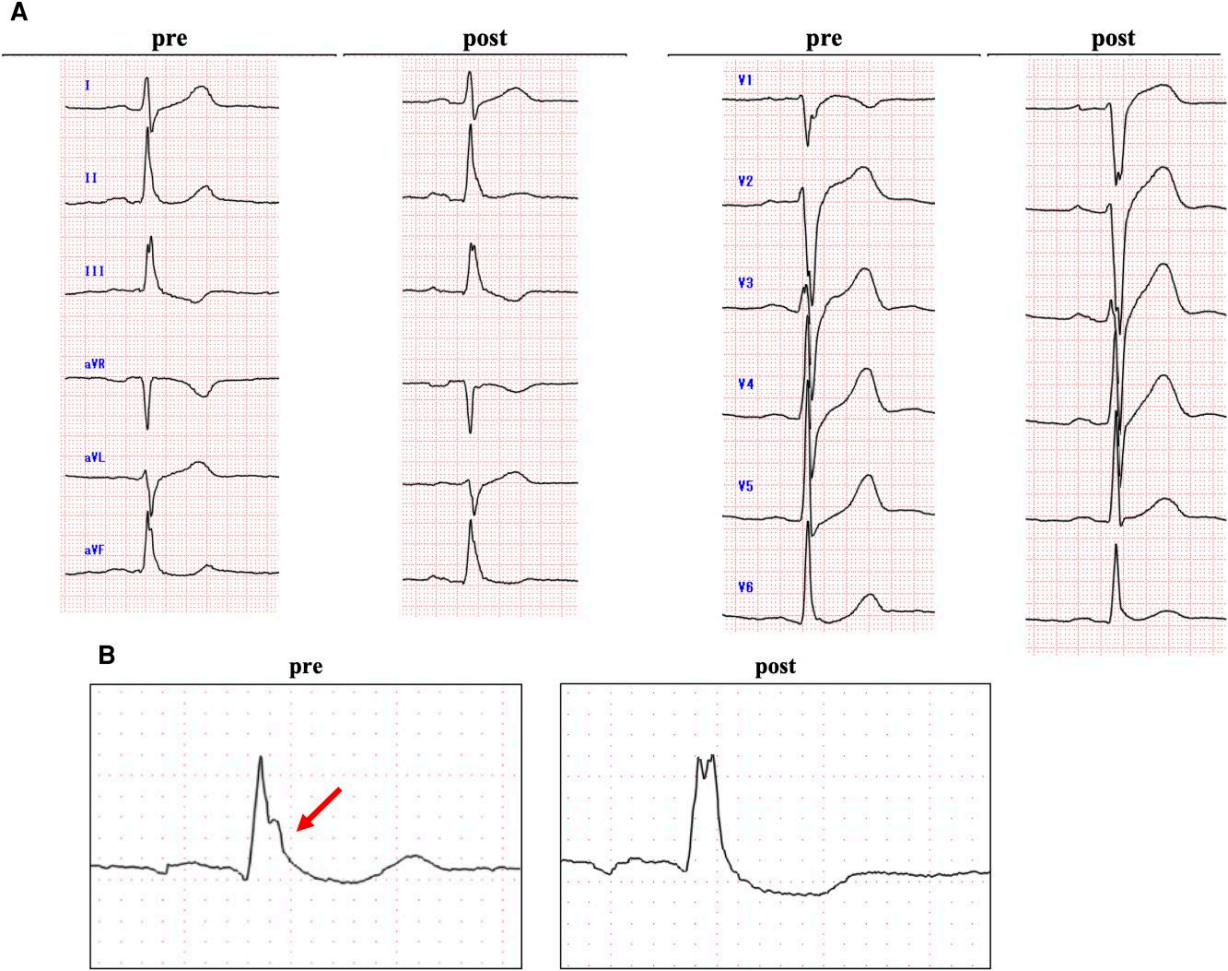
(*A*) Electrocardiogram before and after catheter treatment. The J wave induced by the inferior wall is still present, whereas the reverse J wave seen in the V1 lead disappeared. (*B*) Vertically flipped electrocardiogram in V1 lead. Resolution of the inverted J wave observed preoperatively (arrow).

Following ablation, VF was non-inducible with ventricular extrastimuli, concluding the procedure. Post-procedure pericarditis developed but resolved with acetaminophen and colchicine. The patient was discharged 1 week after the procedure with no arrhythmia recurrence. Follow-up electrocardiography at 1 month showed persistent inferior J waves; however, the inverted J wave in V1 disappeared (*Figure 5*).

Currently, at 8 months post-ablation, the patient has remained free from VF recurrence without antiarrhythmic medications, and no S-ICD therapies have been recorded.

## Discussion

Sodium channel blockers typically accentuate the electrocardiographic and electrophysiological abnormalities in Brugada syndrome.^4^ Patients with Brugada syndrome and concomitant inferolateral ERS display late potentials in RVOT and LV epicardium, which are absent in the endocardium. These abnormalities are accentuated by pilsicainide despite attenuation of ECG early repolarization patterns.^5^ In ERS, pilsicainide attenuates ECG J waves while concurrently augmenting abnormal potentials.^6^

ERS has been classified into two phenotypes based on electrophysiological characteristics. The first *features* delayed depolarization abnormalities predominantly in the right ventricular epicardium, *with* electrocardiographic findings consistent with Brugada syndrome. Depolarization abnormality is characterized by fractionated electrograms with low voltage and prolonged duration. Abnormally delayed potentials are recorded in RVOT and inferior wall epicardium, where they play a critical role in maintaining VF, suggesting depolarization abnormality as the underlying mechanism.^7^

The second phenotype lacks clear depolarization abnormalities, with the Purkinje network serving as the PVC trigger for VF. Ablation targeting the Purkinje network has proven effective in this group.^8^

In our case, pilsicainide enhanced RVOT abnormal potentials despite absent typical Brugada ECG patterns, while paradoxically attenuating LV posterior wall potentials. The earliest PVC activation site was localized near these LV posterior wall abnormalities, with both phenotypic characteristics present. Non-Purkinje PVCs with right bundle branch block morphology originate from LV posterior wall epicardial sites with abnormal potentials,^9^ manifesting as wide QRS complexes with QS-wave patterns featuring intra-QRS notching in lead II.

Ablation of right ventricular epicardial substrates normalizes Brugada ECG,^10^ hypothesized to modify conduction delay from fat or fibrosis. In this case, ablation targeting delayed potentials resulted in disappearance of the inverted J wave in V1. From a vectorial perspective, this inverted J wave may reflect abnormal electrical activity from the anatomically opposite LV posterior wall. Lead V1, positioned over the right ventricular anterior wall, records positive deflections from anterior forces, while posterior wall activity manifests as negative deflections. Abnormal delayed potentials in the LV posterior wall could generate a posterior-to-anterior vector during terminal QRS, resulting in this configuration. Disappearance following LV posterior wall ablation supports a causal relationship between the posterior substrate and V1 findings.

Important limitations exist. First, overlapping ablation procedures prevented sequential ECG assessment, precluding definitive attribution of V1 changes to a specific substrate. Additionally, we cannot exclude minor variations in V1 electrode placement between recordings, though all ECGs followed standard protocols. Separate 12-lead ECGs after each ablation would have determined which substrate caused the inverted J wave. Second, inverted J wave changes following pilsicainide could not be assessed during the procedure. Although pilsicainide attenuated LV posterior wall potentials, corresponding V1 changes were not confirmed, as electrophysiology laboratory monitoring lacks standard 12-lead ECG quality. Had the inverted J wave diminished with pilsicainide, this would have supported the LV posterior wall origin hypothesis. Future studies should incorporate serial 12-lead ECGs at multiple procedural time points. Third, our findings require validation in larger cohorts, as complex electrophysiological interactions in ERS may involve mechanisms beyond current understanding.

## Lead author biography


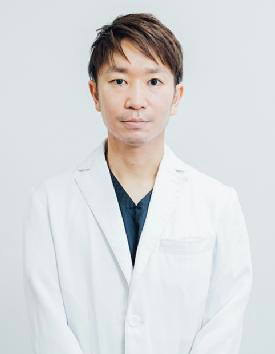


Dr Yasuyuki Takada graduated from Saitama Medical University in 2014. He is currently working in the Department of Cardiology, Tokyo Medical University, in Tokyo, Japan, specializing in arrhythmia disease.

## Data Availability

The data underlying this article will be shared on reasonable request to the corresponding author. Patient data are anonymized and comply with institutional privacy policies.
